# A Qualitative Study of the Impact of HIV/AIDS on Agricultural Households in Southeastern Uganda

**DOI:** 10.3390/ijerph6082113

**Published:** 2009-07-29

**Authors:** Dawn C. Parker, Kathryn H. Jacobsen, Maction K. Komwa

**Affiliations:** 1 School of Planning, Faculty of Environment, University of Waterloo, 200 University Avenue West, Waterloo, ON N2L 3G1, Canada; 2 Department of Global and Community Health, George Mason University, 4400 University Dr. Fairfax, VA 22030, USA; E-mail: kjacobse@gmu.edu; 3 Department of Environmental Science and Policy, George Mason University, 4400 University Dr. Fairfax, VA 22030, USA; E-mail: mkomwa@gmu.edu

**Keywords:** HIV/AIDS, agriculture, food security, sustainability

## Abstract

The HIV/AIDS pandemic threatens economic, social, and environmental sustainability throughout sub-Saharan Africa. This paper reports on a qualitative study exploring interrelationships between HIV/AIDS, labor availability, agricultural productivity, household resources, food consumption, and health status in rural southeastern Uganda. Respondents reported an increase in widow-and-orphan-headed households; labor shortages due to illness and caretaking; degradation of household resources from health-related expenses; loss of land tenure and assets following deaths, especially for widows and orphans; and changes in agricultural practices and productivity. Our study highlights a potential downward spiral of livelihood degradation for vulnerable households and suggests targeted interventions to improve sustainability.

## 1. Introduction

The HIV/AIDS pandemic is one of the most pressing challenges facing rural communities in sub-Saharan Africa. Approximately 63% of people globally living with the virus reside in the region [1] and the most affected households are heavily dependent on agriculture. As a result, the long-term pandemic has eroded the ability of rural African households to produce food and other agricultural products, to generate income, and to care for and feed family members [2,3]. While prevalence rates have declined over time in some regions, and while availability of anti-retroviral drugs (ARVs) has reduced the effects of infection in communities where ARVs are widely available, recent studies have shown that the pandemic continues to exert significant negative economic and social effects in affected areas [4]. HIV/AIDS affects not only the health of infected individuals, but the socioeconomic status of the individuals, their families, and their broader community. In addition to the direct costs of paying for medical care, HIV-affected households experience a loss of labor productivity both from household members who are ill and from their caregivers [5]. In rural areas where farming is the primary source of income and food, decreases in household labor supply can lead directly to reductions in the nutritional status of all household members [6].

Like many other diseases prevalent in sub-Saharan Africa, such as malaria and tuberculosis, HIV/AIDS is not only a burden to infected individuals, households, communities, and institutions, but also creates a severe obstacle to social and economic development and sustainability in many developing countries [7,8], as the resources needed for economic growth are diverted into disease prevention, control, and treatment activities. One of the core Millennium Development Goals (MDGs) agreed upon by leaders from around the world in 2000 is to reduce avoidable death in low-income countries from diseases such as HIV/AIDS, malaria, and tuberculosis [9,10]. Despite this goal, deaths from AIDS-related illness have drastically affected household economies, reducing human capital, agricultural productivity, and labor supply, and in turn reversing progress towards meeting other development challenges [11,12]. There is substantial evidence that households that begin with lower levels of human capital, capacity for agricultural productivity, and available labor are especially vulnerable to falling into a downward spiral of deterioration of household social welfare and livelihoods following HIV/AIDS infection [13]. Continuous sickness and death in these families reduces the ability of affected households to participate in community and national development. Shocks to households and communities from disease events such as HIV/AIDS can reverse development progress, threatening the social and economic sustainability of these systems.

From a more positive viewpoint, addressing HIV/AIDS and its associated socioeconomic problems can reverse the downward spiral of livelihood degradation, propel affected households toward higher incomes, and contribute to sustainable development in affected communities [14]. To achieve this goal, enhanced understanding of the interactions between HIV/AIDS, institutions, agricultural development, and household livelihoods is required. This understanding will not only more clearly reveal the dynamics that threaten the sustainability of the local systems, but will also help to identify which interventions could most effectively halt or reverse downward spirals of livelihood degradation. Potential interventions include food security policies focused on adoption of new marketing strategies and crops, sustainable agricultural practices, health care system interventions, and provision of medical care, social support, and HIV/AIDS education and counseling by local community organizations. Collective research on HIV/AIDS will not only advance HIV/AIDS science, but hopefully will contribute to the development of policies that will help vulnerable households cope with the disease [11]. The goal of our research is to provide stakeholders, policy makers, and governmental institutions with an enhanced understanding of the social effects of HIV/AIDS in rural communities, providing information that will allow for the design of improved policies that integrate successful local-community based efforts with longer-term national and international efforts to enhance the sustainability of these rural agricultural systems.

While the hope and expectation is that medical advances will ultimately eliminate the threat of HIV/AIDS in these communities, the HIV/AIDS pandemic can be seen as one example of how a shock to these rural communities can threaten their sustainability, especially when the communities are home to socially and economically vulnerable populations. Therefore, the lessons learned from this investigation can be generalized to the design of policies that support communities facing related shocks, whether from other chronic diseases, ecological shocks such as crop failure and drought, or economic volatilities such as commodity and energy price fluctuations.

Although Uganda is considered to be an HIV “success story” because the prevalence of infection has decreased from an estimated 15% or higher in the early 1990s, one of the highest rates in the world at the time, to about 6.7% today, there are still nearly 1 million Ugandans who are HIV-infected [15–17]. Most HIV infections occur among adults ages 15 to 49 years old, which causes significant labor losses in the highest productivity age groups [17]. Open communication about HIV/AIDS from both government and non-governmental organizations has helped to reduce the prevalence rate significantly [15,18–21]. Despite the reduction in the prevalence of HIV in Uganda, there is still much to do in order to strengthen the existing programs and to ensure long-term social and ecological sustainability. For example, although ARV use has risen drastically from less than 20,000 Ugandans in 2002 to more than 100,000 in 2007, the drugs are still only being used by about half of the people who would benefit from them [22]. To the extent that many Ugandan communities face challenges due to other diseases, ecological disruptions, and economic shocks, improved policies to mitigate effects of HIV/AIDS may also help to address the broader development challenges faced in these communities.

This paper reports on the results of a series of in-depth semi-structured interviews and focus group discussions conducted in Kampala city and the Mayuge district in southeastern Uganda with government officials, health experts, and local community leaders in 2006. The interviews were designed to gather information regarding participants’ perspectives on the effects of HIV/AIDS infection on household composition, labor allocation, land transactions, health expenditures, and agricultural practices in farming households and communities in Uganda. These interviews are part of a larger, multi-stage research project designed to explore relationships between sustainable agriculture, household food security, and health status in the study area [23,24]. The estimates of household expenses and labor losses and the local rules for inheritance of land and other assets following a household death that are reported in this paper will be used to expand an existing quantitative model that explores the effects of HIV/AIDS on affected households. The interviews reported here also contributed to the development of a follow-up quantitative household-and-individual survey conducted in 2007 that has provided additional data for statistical analysis, cross-validation of the results from this survey, and development of new model rules. The long-term goal of these modeling efforts is to develop a framework to explore the potential effects of various policy interventions, which should contribute to improved policies for sustainable development in the region.

This paper is structured as follows. First, we provide a brief review of the literature on this subject. We then describe our methodology and results. We conclude with a discussion of our observations and how they fit into the larger work that is being conducted on the impacts of the HIV/AIDS pandemic on rural households in Africa.

## 2. Literature Background

**Figure 1.**
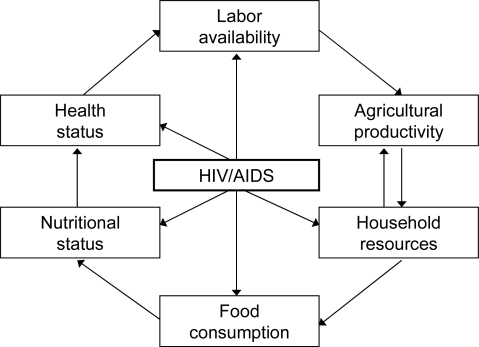
Model of the interrelationships between HIV/AIDS, labor, agriculture, food security, and health.

Figure 1, developed by the authors based on literature review and field work, summarizes the mechanisms through which HIV/AIDS affects household food security, health status, and agricultural production, and illustrates how HIV in a household member can dramatically affect the resources, nutritional status, and health status of the entire household. The central component of Figure 1 is the exogenous event of HIV infection. HIV/AIDS affects many aspects of household socioeconomic status that are illustrated by the components encircling the HIV/AIDS box.

Starting from the top of Figure 1, when a member of a household becomes infected with HIV, household labor will be reduced, both due to the decreased health status of the person living with AIDS (PWLA) and through the labor requirements for caretaking. Loss of labor will result in decreased agricultural productivity. Household resources are likely to decline due both to increased HIV related expenses and to decreases in the value of agricultural production. These reduced resources may lead to further reductions in agricultural output as production-related capital is depleted. Reduced household resources may also decrease food consumption at the household level, which is particularly damaging because of the potentially increased demands for highly nutritious foods for the PLWA. Decreased nutritional status can, in turn, potentially result in reduced health status for both the PWLA and other members of the affected household. Illness and fatigue lead to additional reduced labor productivity. Thus, this figure summarizes the potential dynamics in an affected household that can lead to a downward spiral of livelihood degradation.

There is a growing literature that explores the relationships shown in Figure 1 and describes the effects of HIV/AIDS on smallholder farming, socioeconomic status, the nature and management of the household labor force, agricultural activities pursued, and cropping patterns [3,25]. Below, we review that literature to identify evidence related to the effects of HIV/AIDS on household composition, labor allocation, land rights, social status, and welfare; on agricultural production, including crops grown, total area cultivated, total production, and agricultural income; and on participation in local markets.

***Household composition and structure.*** Most research on HIV/AIDS has focused either on the household head or on the PWLA as the unit of analysis, but for studies of agricultural production, the household is generally considered to be the relevant unit of analysis. In sub-Saharan Africa, households account for the majority of agricultural production, agricultural production is the main source of self-employment, and the majority of households rely on household labor for agricultural production [26]. Many authors stress that the effect of HIV/AIDS-related illness and mortality depends on which members are affected and on the demographic structure of the remaining household members, since roles in household and agricultural production and inheritance and property rights are age and gender specific [27].

The loss of a household member due to HIV/AIDS has led to widespread changes in household structures [2], including an increasing number of widows and widowers, increases in female or orphan-headed households, and the break-up of families [26]. Traditionally, orphans are taken in by the extended family (uncles and/or grandparents), potentially supplementing household labor but also increasing household consumption needs, financial burdens due to tuition costs, disruption of children’s income earning opportunities, and dependency ratios [28–32]. Increasing numbers of orphans have created a crisis of orphan care [33].

***Labor availability and allocation.*** In sub-Saharan African agricultural households, the family provides the majority of support and care for the PLWA [26]. During the AIDS patient’s sickness, most of the health care is done by women, including wives, mothers, sisters, daughters, aunts, and grandmothers [26,34]. In most countries in the world, caring for the sick is considered to be a woman’s task, while grandparents also traditionally accept the responsibility of caring for their grandchildren [30,34].

Agricultural production requires high inputs of physical labor and technical skills. HIV/AIDS mostly affects young adults, usually the most active and productive group of the society, and this greatly affects the availability, quality, and human capital of the agricultural labor force [1,35–37]. When possible, affected households may bring in additional labor through new family members or hired labor to compensate for lost production, but many households do not have the resources to recruit additional labor.

However, decreases in agricultural labor and agricultural productivity have not been identified in all studies from sub-Saharan Africa, although these findings may be due to limitations in study design. For example, Beegle [38] found relatively few differences in hours allocated to farming and chores for household members, both preceding and following the death of a household member in the Kagera region of Tanzania. Onyango *et al.* [39] found no significant changes in labor hours between illness-affected, death-affected, and non-affected households, although they did find that labor productivity was reduced by 52 percent and 65 percent for death and illness affected households, respectively.

In some countries, including Uganda, labor shortages may intensify as a result of extensive mourning, during which the bereaved do not participate in agricultural production and other economic activities [30,35,40]. During mourning, less time is allocated to agricultural activities, resulting in untimely weeding or late planting and reduced agricultural output.

***Land tenure and land transactions.*** Recent case studies have also revealed the impact of HIV/AIDS on land tenure. In rural Africa, land is considered to be an important asset that will sustain the livelihood of future generations, and it is the main generator of income through crops, livestock, and/or rental to others [30]. Affected households are more likely to lose their land than their counterparts, with land holdings being transferred to in-laws, clan members, or creditors [41]. Land inheritance rights in sub-Saharan Africa generally follow the male lineage. Thus, widows and orphans often lose rights to land tenure, with the late husband’s extended family claiming land and other assets [35,42]. While land is frequently intended to be held in trust for male orphans by male caretakers from the extended family, land is not always returned when the oldest son comes of age [35]. Widows may also be reluctant to rent out land to others for fear of losing land tenure rights [40]. HIV/AIDS may exacerbate tensions over property ownership that already exist because of population pressures, poverty, and gender inequality [42].

***Social status and social connections.*** In many societies, AIDS is seen as a shameful disease, and PLWAs experience stigma that results in reduced participation in community life, exclusion from social networks, and less access to information, education, counseling, and other support services [42–44]. Stigma can affect not only the individual PLWA, but also the entire household. PLWAs and their family members may withdraw from participation in agricultural clubs, where information on new agricultural technologies is often shared, and this may reduce the household’s knowledge and farming skills. In addition, lack of participation in agricultural clubs reduces engagement in wider debates on community development that influence policy processes and other broader agricultural objectives. HIV-related stigma may also limit food marketing opportunities and interactions with agricultural extension workers, and may decrease participation in community development activities [44,45]. In short, the critical support provided by social and economic networks may decrease at the times when it is most crucially required.

***Household health expenses.*** Households may expend substantial resources caring for a PLWA through all stages of illness [35,38,46,47]. These expenses may reduce the household’s available capital, since cash is required for drugs, health care, hospital stays, and special highly-nutritious foods that are recommended to be eaten with HIV drugs [26,35,39]. The burden of direct health care costs, transportation costs, lost time and wages due to waiting at health centers, and side effects can reduce adherence to antiretroviral drug regimes and contribute to negative treatment outcomes [48]. Deaths are generally associated with substantial expenses, including funeral ceremonies, burials, and potentially even travel and support costs for mourners [38,39]. A death may further increase demands for cash and erode the remaining household income, inducing sales of livestock, farm equipment, and other assets [3,49]. Despite these demands, survivors will try to protect valuable assets such as land and trees to ensure the survival of the household. Non-affected households may also provide assistance to members of other households after the death [38]. However, reductions in household incomes are found for HIV-affected households, particularly for poor and female-headed households [50]. Children may also be taken out of school to supplement the household labor force and/or due to lack of funds for school fees [3], further eroding human capital in years to come.

***Cropping patterns.*** The reduction in available labor and household resources associated with HIV frequently induces changes in cropping patterns for affected households. Changes from more intensive cash crops to extensive crops that require lower levels of labor, fertilizer, and herbicides and more flexible planting schedules and labor requirements have been found in countries throughout eastern and southern Africa [2,37,39,40,50–56]. However, it is important to note that many factors that affect household incomes and create uncertainty about market prices may induce shifts in cropping choices. For example, the shift from maize to cassava in Malawi was also likely influenced by the removal of fertilizer subsidies in 1992, since purchased fertilizer is essential to maintaining agricultural productivity in depleted soils, but is one of the most costly cash inputs for poor farmers [57]. Other reviews have not found a higher production of subsistence crops by affected households, and instead attribute reductions in maize and other cereal production to economic factors [58].

***Agricultural production and cultivated area.*** Beyond changes in cropping patterns, reductions in household labor and resources can be expected to lead to reductions in agricultural cultivation and output. With less labor, household farms may be reduced to a more manageable size or left to fallow [6,53]. In other cases, the time demands of caring for the PLWA may lead to delays or to skipping of weeding, tillage, or planting, further reducing agricultural productivity [2,3,45,59]. The death of an adult male head of household is particularly associated with declines in cultivated area, since adult males are generally responsible for land clearing and cultivation [2,6,39]. Beegle [38], citing reductions in the production of food crops (maize, cassava, and beans), makes the important point that reductions in agricultural output may be expected following a death, since household nutritional and subsistence needs have fallen. This hypothesis makes sense for subsistence producers, but may not hold for those who produce for the market and purchase food. Other authors report reductions in agricultural output and land productivity, especially following the death of a male head of household and for poorer households [39,40,60].

***Market participation.*** Demands for caretaking and the need to replace the lost labor of the PWLA can theoretically reduce off-farm and income-generating labor activities. Women are likely to have income-earning activities curtailed due to care-giving responsibilities [49]. Affected households may reduce off-farm labor supply in some cases, but may also hire other unskilled labor to replace lost household labor in others [50]. Beegle [38] found significant reductions in participation in non-farm self employment and wage employment for adult men in the six months preceding a death.

***Differential vulnerability and food security effects.*** The range of literature reviewed demonstrates how HIV/AIDS adversely affects household living standards and how structural changes within the household system result in a loss of agricultural production. Actual household impacts will be highly dependent on (1) the age and position of the affected household member, with the death of an adult male head of household potentially having the most financial impact, and the death of an adult female negatively affecting children’s educational opportunities and participation in household labor; (2) the financial condition of the households, with poor households being much more vulnerable; (3) the type of agriculture practiced in the region; (4) the current limiting factors (land, labor, or capital) in the agricultural systems; and (5) whether formal markets for labor and agricultural outputs exist, and the extent of participation in these markets by households [6,40,61,62]. Reductions in household agricultural output and income and changes in cropping patterns can reduce household’s food security [63], reducing the nutritional quality of the diet [3], and decreasing the number of meals per day [41].

## 3. Methods

**Figure 2.**
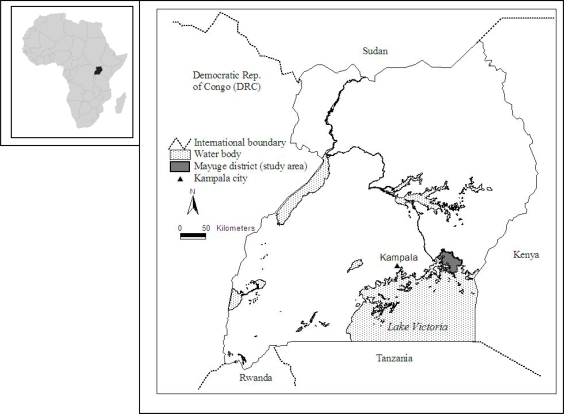
Map of Uganda showing the Mayuge district (study area).

***Study Area.*** Our study area is the Mayuge district in Southeastern Uganda (Figure 2). The Mayuge district lies at an approximate altitude of 1,070 meters above sea level, and the district has a bimodal rainfall pattern that ranges from 1,250 to 2,200 millimeters per annum [64]. As many people in these study sites depend on subsistence agriculture, most crops are grown in mixed farming systems. These crops are grown on small parcels of land (0.5 to 4.1 hectares). The main food crops include bananas, maize, cassava, sweet potatoes, rice, beans, fruits and vegetables, and cash crops such as coffee and cotton. Fruits and vegetables are usually grown around the homestead (kitchen garden). Additionally, some households in the district sustain their livelihoods by raising livestock, fishing on Lake Victoria, and through employment at sugar plantations in an adjacent district. While official HIV prevalence rates are not available for the study area, the government of Uganda estimates that about 5 or 6% of rural residents have HIV infection [22]. Health facilities are separated by distances greater than 2 km, hindering accessibility for many groups.

***Qualitative research methods.*** The qualitative semi-structured research methods used for this study are often incorporated as part of a multi-stage research process that combines both qualitative and quantitative methods of investigation. Our interviews had two goals: to gather informed local estimates for several factors related to the impacts of HIV on local households in order to parameterize a simulation model, and to inform the design and development of a follow-up quantitative survey. In such research, often a limited number of informants are selected intentionally, so that their position, experience, and extensive knowledge can enhance the understanding of the issues under investigation [65,66]. Following this strategy, our informants were drawn from various backgrounds designed to represent a cross section of national, regional, and local experts and local stakeholders both directly and indirectly affected by HIV.

***Participants.*** Extensive work was conducted prior to the fieldwork to identify interview participants. Letters were sent out to AIDS support and advocacy organizations, the Uganda Ministry of Health, the STD/AIDS Control Programme, and government officials in Mayuge District. A local fieldworker was recruited through contacts at the USAID Micronutrient Program (MOST). The fieldworker, who was familiar with the local communities and language and had prior experience in field research, followed up with the contacted organizations to arrange an interview schedule. Interviews were first conducted in Kampala (the capital city) to gain information on the impact of HIV/AIDS both nationally and within rural communities. Interviews were then conducted in the Mayuge district. In Kampala, representatives from five HIV/AIDS support NGOs were interviewed. In Mayuge, we interviewed three district officials, two government agricultural officers, four district and local level health officials, seven local and community leaders, ten NGO representatives, one leader at a community development organization, and four local farmer representatives. In addition, four focus group discussions with seven to ten participants in each were conducted.

Our informants fell into three general categories: employees of non-governmental organizations (NGOs), community members, and government officials. NGOs are the major providers of HIV voluntary counseling and testing (VCT) training in Uganda and also provide medical care, social support, and HIV/AIDS education programs to their enrolled members. The trained counselors and staff members we interviewed provide services to rural communities, both in the form of direct support for AIDS affected households and through general development assistance in areas such as agricultural and economic development, health, hygiene, sanitation, food security, and sustainable agricultural practices. The community members we interviewed included representatives from households directly affected by HIV/AIDS and from households that did not have a current or former household member with the disease. This was essential for getting a diverse set of perspectives on the impact of HIV/AIDS on agriculture and livelihoods in communities in the Mayuge district. Finally, the perspective of government agricultural officials and community leaders reflected their experience working with diverse sectors within their entire communities. Therefore, we believe that our pool of interviewees reflects the broad perspective of the community.

***Interviews.*** Interviews were conducted from May 29 to June 10, 2006, in the language preferred by the participant (transcripts of interviews in local languages were translated into English before analysis). Each interview was conducted at a meeting place identified by the participant as convenient, usually a workplace, church, or public outside meeting area. Individual interviews were 45 minutes in duration, on average, and focus group discussions were held for about 60 minutes each. Two trained local research assistants helped with the interviews, transcribed responses, and recorded observations about focus group participants.

Participants were asked to discuss the impacts of HIV/AIDS and related illnesses and deaths on households in their communities. Questions were asked about household structure and activities, agricultural production (crop patterns, labor allocation, and land transfer), and labor markets. Additional questions were asked about the livelihood/farming systems in their communities. (A summary of the interview guide for these conversations is provided in Appendix A; the original question ordering and wording are available on request.)

***Ethical considerations.*** Participants were not paid, but were offered a free soft drink during the interview or focus group. Ethical approval for the study was granted by both George Mason University (Fairfax, VA, USA) and the Uganda National Council of Science and Technology (Kampala, Uganda). All participants provided written informed consent.

## 4. Results

***Household composition.*** Study participants indicated that new household structures are gradually emerging in their communities as the proportion of households headed by widows and young children increases. In the past, widows were usually taken into the household of a male relative (clan member) of her husband as a wife, but interviewees report that this practice is losing its popularity due to fear of contracting HIV from the widow. Child-headed households are also increasing in number as eldest children—usually sisters—take on care of their orphaned younger siblings. Decisions about the future of widows and children following the death of a male head of household are often made by male relatives, in some cases in consultation with the local community leaders. Several respondents noted, however, that this traditional method of decision-making is being at least partially replaced by wills that specify the desires of the deceased family member.

***Labor allocation.*** The majority of participants reported that AIDS has affected the number of workers available for agricultural activities in their communities. The reductions in female labor due to both HIV/AIDS in women and the increased demands for time-consuming care giving activities are especially important in reducing agricultural production, since women are responsible for most agricultural activities. Estimates of the reduced work hours for both PLWAs and their family members who serve as caregivers were developed based on estimates of work hours for affected and unaffected household members provided by study participants from the “labor allocation” questions. Using local terms for the progression of HIV/AIDS, PLWA in “stage A” (which corresponds with stage 1 of the World Health Organization’s classification system and is the asymptomatic stage following initial infection) were estimated to work only 25% of their pre-diagnosis work hours, PLWA in “stage B” (which encompasses WHO stages 2 and 3, the early symptomatic stages) were estimated to work only 10% of their pre-diagnosis hours, and PLWA in “stage C” (WHO stage 4, clinical AIDS) contribute no work hours. Furthermore, there is a reduction in the hours of work by caregivers, as estimated in Table 1.

The reported work hours of PLWA, especially those in stage A and even stage B, seem very low, especially given that many households in the area where this study was conducted are engaged in subsistence agriculture and are food insecure even when all family members are contributing labor to the household. These reported work reductions likely reflect a perceived “ideal” work schedule rather than reality. Interviewees reported that HIV/AIDS counselors and health care officials in the community advise people with HIV infection to limit their work hours and to not overexert themselves, even if they are feeling healthy. Respondents shared the belief that if possible a person with HIV/AIDS should “rest more” and “conserve energy” in order to promote a longer life. For example, a local community representative commented:

The affected household may work daily but the time is somehow shortened because they also have to care for the sick person. Some people start digging at 7am and return at noon. But when they have a patient they have to prepare breakfast so they leave at 8 or 9am and return at 11am to prepare meals and care for the sick. In most cases they do not return in the afternoon. For the infected person, there are those who have joined organizations like TASO [a community-based AIDS support organization]. With help of advice they are boosted and get energy. Some of them work normally but they are also advised not to overwork. Some work from 7 to 9am but they do not return in the afternoon. In that case they can do other house activities.

Respondents also noted that few households can actually afford to observe these guidelines, and indicated that they knew of few people who had actually been able to cut back on work when not seriously ill. An agricultural community coordinator commented:

The PLWA usually does now work but it depends. If he is the head of the house he is forced to work. Mostly in a week the person can work for 5 days otherwise if he/she works too much the disease will bring him/her down.

However, even if the labor reductions are not as severe as reported, respondents expressed concern that care giving requirements have significantly impacted the economic security of most HIV-affected households.

Follow-up questions about work involvement in particular activities revealed slightly less drastic reductions in labor availability, but also raised concerns about the time required for seeking medical care. PLWA were estimated to work on the farm or in the garden a little less than half of the hours worked by healthy family members and to contribute only a few hours a day to completion of household chores. PLWA were also estimated to contribute less to “heavy” labor activities. Some of these work hours were replaced by the considerable amounts of time estimated to be spent traveling to clinics for medical care. The time required for clinic visits was estimated at 5 hours a day one day per week during stage A and two to three days per week during stage B. During stage C, the burden of time seeking medical care shifted to a family member, who was required to pick up medications once the PLWA became bedridden.

***Land transfers***. Land transfers following the death of a parent or husband are a major concern in Uganda, where clans control most land and individuals have limited rights. Participants indicated that the majority of men distribute their land to their adult male children before they die. When male children marry, they bring their wives into the household. Therefore, rules that ensure that male children will inherit land serve to keep the land holdings of a particular clan concentrated geographically. However, these inheritance practices can leave widows without land resources and livelihoods, unless a widow with minor children is allowed to remain on the land until a son comes of age. Children who are orphaned might have the land transferred to an uncle or other male relative at least until the oldest male child comes of age; however, some widows and orphans are left without any land after the death of the male head of household. Respondents emphasized their concerns in comments like these from NGO representatives:

It is obvious that the male progeny takes responsibility as heir. The land is kept in the family; a brother takes over if there are no sons. Women are viewed as property and part of the package. So in this case daughters are not regarded as part of the family or clan. Only sons are seen as heirs and so socio-culture does play a role in land transfer following death of a man in the family.

The land is normally passed on to the boys and their families while the girls mostly get nothing. This has to do with socio-culture because girls are not recognized. For widows, they are not recognized either, and this is a problem. In the land tribunals these are the most frequent cases. If the widow doesn’t have children, there are no discussions; she has to pack her belongings and go.

The rules are changing to some extent in an attempt to protect the inheritance rights of women. For example, there are now community members called “*obutaka*” who help to settle clan disputes and help widows to protect their land rights.

**Figure 3.**
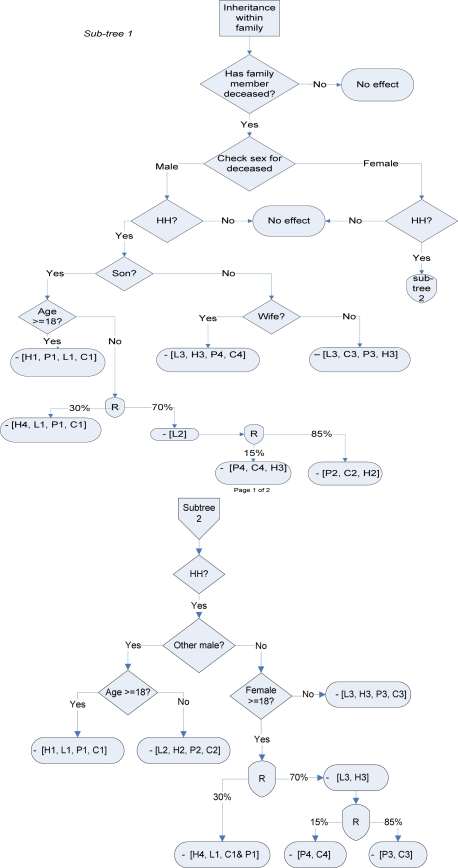
Model of the interrelationships between HIV/AIDS, labor, agriculture, food security, and health.

Figure 3 summarizes land transfer rules based on our qualitative interviews. As indicated earlier, one goal of this qualitative survey was to develop modified decision rules for an existing simulation model. Detailed decision trees that formalize the land and property inheritance rules in the study area were developed based on the results of the qualitative study. The flow charts identify the person designated as the new head of household and trace the transfer of household assets (land, household members, and livestock) following a household death. Sub-trees 1 and 2 illustrate rules for a male and female death, respectively. In cases in which respondents indicated that multiple outcomes were possible, we coded multiple outcomes with estimated probabilities of each. For example, in this chart, we assume a 30% chance that a remaining female will be able to keep her household assets and become the new household head. Alternately, if the assets return to her husband’s clan, we assume an 85% chance that the clan will take the remaining household members into its household, and a 15% chance that the woman will be expelled and will return to her household of origin. These assumptions could be varied in a simulation model to explore the potential effects of policy interventions aimed at strengthening the inheritance rights of women.

***Family structure.*** Participants raised special concerns about the fate of children as the number of orphans overwhelms the number of available caregivers. In traditional African culture, children are members of an extensive network of relatives beyond their biological parents. Community leaders expressed fear that the traditional extended family of Basonga culture is being lost as the demand for orphan care increases. In the past, the death of both mother and father was a relatively rare event, and the extended family could afford to take in a few orphans. The AIDS epidemic has greatly increased the need for orphan care, and has also caused economic hardships that prevent extended families from taking on the responsibility of raising additional children. Also, community leaders noted that a greater emphasis is being placed on independent nuclear families, and that is contributing to the breakdown of traditional support networks. Thus, even though relatives are often willing to provide some support for children in their extended families, perhaps by providing some financial support for child-headed households, responsibility for support of orphans now often falls beyond the extended family. Although many non-governmental organizations are now operating in this community, support is minimal and need is growing.

***Social status and social networks.*** Participants indicated that the perception of PLWAs in their communities is changing. More women are joining support groups and doing alternative income generating activities. Furthermore, individual respondents and focus group participants indicated that new community-based groups are involving both non-affected and affected people as a way to empower the whole community. Several organizations and events have been initiated specifically to fight stigma and discrimination against the PLWAs in the community. The AIDS Support Organization (TASO), an NGO that provides a range of supportive activities for members, encourages the formation of drama groups as a tool for AIDS awareness in the community (counseling and testing for HIV are required in order to become a member of TASO). However, the potential for stigma and evidence of its effects still exist. This is indicated by the fact that many men hide their status from their wives. In some cases men will go to different counseling agents or will buy drugs far from their villages, where they think their records will remain confidential from their spouses and other community members.

***Health expenditures.*** It is difficult to obtain estimates for the actual costs of medical care for PLWAs because of the long duration of illness. Survey participants were unable to give exact estimates for these expenses, but emphasized the great variety of costs incurred by PLWAs and their families, including transportation to clinics, laboratory tests, clinician and hospital fees, medications, and funeral costs, as well as the purchase of special nutritious foods recommended by TASO. Although medications are supposed to be available at no cost at government hospitals, supplies are often low, which requires PLWAs to purchase subsidized medication from non-governmental organizations at a cost of about 100 Ugandan shillings per day (roughly 5 U.S. cents at the time of the interviews). Additional expenses are incurred by visiting traditional healers, transporting the body for burial, and paying for food for relatives who travel to visit the PLWA and those who attend funeral services and stay for several days or weeks after the burial. Costs for funerals are managed to some extent through community organizations that provide a form of funeral insurance. Most participants indicated that there are a growing number of households that are unable to meet the expenses of the illness and death of a family member, and that a growing number of PLWA can only afford panadol (acetaminophen) or aspirin for pain management rather than drugs specifically for HIV/AIDS. Table 2 illustrates estimated food and health expenses by a household that is affected by HIV/AIDS.

These illness-related expenses are clearly eroding household livelihoods, especially of poorer and more vulnerable households, as summarized by the following comments from community leaders and NGO representatives:

Medication increases expenditure during illness. People divert the little funds they have to buy medicine. Sometimes we do meet young people and on many occasions they mention that they are orphans and had sold land to take care of the sick. In some situations people sell their houses and forgo their savings, but I don’t have concrete figures.

When the people come to see the sick, the family members have to prepare for the visitors. They hardly come with food. The visitors just come to greet the sick person; they do not help with agriculture activities. This feeding means using the little they have. By the time a person is sick for a year or two, the family is left with nothing.

The bills of medication go beyond death. Sometimes these are higher than the income of the family. People have died in hospitals and have associated costs including the funeral rites. During the funeral, in order to feed the crowds, they encroach on the property and land and animals are sold off to meet the expenses.

TASO works with the Ministry of Health to provide resources to families that are affected with HIV/AIDS, including supplementary non-perishable food. This contribution offsets the expenses listed in Table 1. Table 3 shows the supplementary food that is typically given on a monthly basis by TASO to HIV-infected members.

***Changes in agriculture.*** Respondents consistently reported that agricultural production has gone down in all affected households over the past five years. Reasons reported included the time demands of caretaking and the limited ability of the PLWAs to perform agricultural activities such as digging, planting, weeding, and timely harvest. There was, however, general agreement that plant diseases such as banana and cassava mosaic and extreme weather events might have contributed to the low production in addition to the HIV epidemic. Households with multiple wives (a common practice among the Basonga people) and many children usually maintain their usual farming practices, and households with greater financial resources may hire additional laborers so that they can grow their preferred crops. However, even in light of general population growth—and a resulting increase in the number of individuals seeking to cultivate the same land area—respondents noted that affected households may leave some land fallow because they do not have time and labor to devote to agriculture.

Study participants noted several changes in agricultural practices that have occurred in households affected by HIV/AIDS. While changes to crops with lower labor requirements were reported, in many cases, intensified production of market crops (such as high-yield varieties of bananas, maize, and cassava, poultry, vanilla, and aloe vera) was also reported. Adoption of many of these crops was supported by the National Agricultural Advisory Services (NAADS) and the outreach programs of local community support organizations through programs designed to help rebuild the livelihoods of small scale subsistence farmers and other vulnerable households that lacked adequate household labor for production of other market crops. This more intensive production allowed households to gain higher yields for lower levels of labor input and cultivated land. Production of poultry, vanilla, and kitchen gardens can occur close to the home, minimizing travel for caretakers and PLWAs. Adoption of labor-saving and output-enhancing production techniques such as use of manure to improve fertility and use of grass around contour bands to reduce erosion have increased for households able to make these innovations. However, some shifts from cash crops to subsistence crops were also reported. Another concern is that diseases like banana and cassava mosaic and extreme weather events can lead to low production, and any reduction in output can push vulnerable households into hunger.

***Agricultural market participation.*** When asked about their ability to sell their goods in remote markets, participants reported that due to high time-cost of illness, affected households may be less able to transport their goods to far-away markets, such as those near the Kenya-Uganda border, where they might obtain higher prices for their products. They may, therefore, sell their produce to middlemen who come to buy produce cheaply in villages and sell them in town at higher prices. Participants believe that these farmers are being offered unfairly low prices by these middlemen, who are exploiting the vulnerability of affected households. This problem has inhibited the success of the backyard vanilla production program in particular. A potential solution to this problem is the formation of marketing cooperatives that may allow farmers to obtain higher prices and to avoid the middlemen. A village leader’s comments reflect this perspective:

The market is a problem to the farmer. The farmer is being exploited by the middleman. We don’t need middlemen and perhaps through organizations like Uganda Farmers Association we can have a stronger voice.

## 5. Discussion

***Household composition.*** Although we did not find evidence of higher female mortality, our general results are quite similar to other studies, with a reported increase in female, elder, and child headed households. This often results in new higher-responsibility roles for more vulnerable family members (young household heads and female children), who were often reported to leave school and/or enter the labor market, and even migrate to ensure their own survival and that of the household. It is also clear that a crisis of orphan care is occurring.

***Labor allocation.*** As widely reported elsewhere, our results indicate that caretaking responsibilities fall most heavily on women and girls, and our quantitative estimates indicate that these responsibilities can absorb the majority of the caretaker’s time as the illness progresses, representing a substantial loss of labor for the household. Our work-hour estimates demonstrate that women consistently work a higher number of hours than men, regardless of the type of work activity. Consistent with other studies, household and agricultural labor is also lost due to the PLWA’s lowered ability to work. We found that in some cases these labor reductions are based on advice of medical professionals, and are an attempt to preserve health status. We also found that these voluntary work reductions may not be possible for poorer households, potentially contributing to a downward spiral of deterioration of health along with increased poverty.

***Land transfers***. Consistent with many other studies, we found that women and girls are highly vulnerable to loss of not only land, but also household capital, and in some cases even the loss of their home within a given community. As shown in Figure 2, widowed households without a male child who might retain rights to the land are most vulnerable to loss of land. While the traditional land tenure system is an institutional structure that has evolved to maintain control over scarce land resources within the extended family unit (clan), it is clear that this traditional structure substantially increases the vulnerability of female-headed households. The existence of community organizations whose goal is to protect the inheritance rights of women is a significant development, and the relative success of these organizations will likely have a significant impact on the well being of affected households and potentially on the distribution of land holdings within the community.

***Social status and social connections.*** Relative to other studies, we find a somewhat more optimistic picture regarding social stigma and loss of social connections because of HIV/AIDS. In Uganda’s case this may be related to government policies and information campaigns, such as the “Zero Grazing” policy implemented in the 1990s that evolved into the current “ABC” policy (Abstinence, Be faithful, Condom use), that have helped Uganda maintain a relatively low HIV prevalence compared to other countries in sub-Saharan Africa and have encouraged open discussion about the disease [20,67,68]. While there is evidence that many men still attempt to hide their HIV status, the development of community-based organizations that provide both social and material support has increased incentives for citizens to disclose their HIV status. These organizations also offer community-based participatory solutions to some of the problems brought about by HIV/AIDS, potentially further reducing the stigma and isolation of the disease.

***Household consumption and health expenditures***. However, consistent with other studies, we find that expenses from HIV have a severe negative effect on household welfare. These effects seem most pronounced in poorer households, which lack resources even before having an HIV-infected household member. These effects are seen both during illness, when households cannot afford drugs, other medical care, and appropriate foods, and also after a death, when they incur funeral expenses that remain constant regardless of household resources. In contrast to other studies, we found that the importance of consumption of highly nutritious foods was highly emphasized, and purchase of these foods often seems to take priority only over the purchase of ARVs. Both governmental and nongovernmental organizations were reported to be providing both food and medical support, but access to both seemed uneven and somewhat unreliable. Our results provided more qualitative detail regarding potential in-kind contributions from relatives. We found a mixed picture, with assistance being uncertain and potentially declining over time. Again, as in the descriptions of increasing burdens of care for orphans and the resulting increase in the number of independent nuclear households, we see evidence of a support network stretched to its limits, and of households needing to put a high priority on meeting their own needs rather than supporting more distant relatives.

***Agriculture.*** As reported elsewhere in sub-Saharan Africa, declines in agricultural production were also reported in our study area. Our study finds a mixed picture in terms of cropping patterns. Consistent with other studies, shifts to crops with lower labor requirements are reported [37,40,52]. However, this shift did not always mean a shift towards a subsistence crop. While the increase in female-headed households may be associated with an increase in subsistence or food crops, this may be due to a cultural preference and/or a higher weight placed on household subsistence requirements rather than lower labor availability [69]. In fact, in contrast to other studies, our respondents reported intensification strategies that often included switches to higher-valued market outputs, such as vanilla, aloe vera, and poultry production. These strategic crop changes appeared to result from lower labor availability and, in some cases, from a subsequent reduction in cultivated area. Consistent with other studies, what cropping changes were reported seemed to depend on household resources, with respondents reporting that households with sufficient labor and financial capital were less likely to change cropping patterns.

***Participation in markets.*** We did not find other studies that discuss the effects of HIV/AIDS on land rental and sales, other than the indication that women may not have rights to sell land, and may be afraid to rent land for fear of losing title. Our specific questions regarding land transfers led us to identify two important potential effects. First, a PLWA who is a household head may try to acquire additional land to provide an inheritance for his children. Second, consistent with other studies, we found that households will sell land only as a very last resort, since in this land-scarce region it is a highly valuable asset.

Although we did not investigate labor market interactions to a great extent, our survey provided evidence that those able to hire outside labor were more likely to be able to maintain their previous production strategies. Rather than finding that household members who previously participated in external labor markets returned to the household to supplement household agricultural labor, we found the opposite, with women and children sometimes forced to leave agriculture and seek external wage employment. Our findings related to crop marketing and the effects of illness on participation in remote markets are very consistent with other studies. The greatest marketing concern raised was a vulnerability to exploitation by middlemen that is being solved in part through the development of community-based institutions (marketing cooperatives).

## 6. Conclusions

Our qualitative findings have shown how detailed qualitative research can bring insights into complex issues surrounding HIV/AIDS in the rural communities of Uganda. They have provided insights into the social and economic burden posed by a household member infected with HIV through the progression from early infection to AIDS to death, and they have provided some preliminary insights into the strategies through which some households cope with the challenges faced. Results from this study were used to guide the development of a follow-up quantitative survey. In particular, these fieldwork results helped to identify significant new issues (voluntary labor reductions, the importance of “special foods,” and issues related to gender, household status, and land transfers) that resulted in development of an important set of quantitative follow-up questions. The research therefore also illustrates the complementarity between qualitative and quantitative research methods. At the same time, these interviews highlighted the many other development issues facing these communities. For example, productivity declines in agriculture were said to occur both due to the effects of HIV and to shocks to the agricultural system. The semi-structured interview format helped to reveal additional potential threats to household livelihoods, thus potentially avoiding incorrect conclusions or recommendations based on a more narrow understanding of the issues faced by these communities. At the same time, the research reveals that particular policy interventions, such as helping households develop a more resilient portfolio of agricultural activities, may help protect vulnerable households against multiple shocks.

As shown in other studies, there are significant obstacles to sustainable development in communities that are highly affected by AIDS as the resources in these communities are being diminished [70]. Households that lack secure rights to land and other property and have low levels of household labor and assets appear most vulnerable to loss of food security, descent into poverty, and even the collapse of the household. These findings imply that policy interventions that directly address these vulnerabilities may be most effective within these communities.

We believe local communities should be involved in identifying and designing appropriate and effective programs to address local challenges. These communities should work not in isolation but hand in hand with NGOs and the government to address these concerns. Such multi-scale, multi-actor policy approaches can increase the prospects for a longer-term sustainable environment for these vulnerable groups. While information such as that provided by our in-depth interviews can in and of itself contribute to improved policy design, the quantitative simulation models developed through the larger project of which these interviews are a part should also contribute to participatory opportunities for exploration of alternative outcomes and policy design by stakeholders at multiple levels.

## Figures and Tables

**Table:** Key for resource distribution outcomes from Figure 3.

Category	Outcome	Category	Outcome
Land	L1: No change L2: Temporary shift to clan (until male = 18 years old) L3: Permanent shift to clan	Livestock	C1: No change C2: Temporary transfer to clan C3: Permanent transfer to clan C4: Divide 25% to 75% to oldest female; rest to clan household
People	P1: No change P2: All go to clan household – temporary (until male = 18 years old) P3: All to clan household – permanent P4: All to other household (wife)	New household head	H1: “Son” (next eldest male) H2: “Clan” – temporary new household H3: “Clan” – permanent H4: “Eldest wife” (oldest female) – permanent H5: “Eldest wife” – temporary (until male = 18 years old)

**Table 1.** Estimated reductions in labor contributions by caregivers of PLWAs.

**Stage of illness**	**Male PLWA**	**Female PLWA**	**Child PLWA**
Stage A (WHO stage 1: asymptomatic HIV infection)	None	None	No more than required for a healthy child
Stage B (WHO stages 2 and 3: symptomatic HIV infection)	50% reduction in labor for a wife or female child	50% reduction in labor for another wife, female child, or husband	50% reduction in labor for a mother or sister
Stage C (WHO stage 4: clinical AIDS)	85% reduction in labor for a wife or female child	85% reduction in labor for another wife, female child, or husband	85% reduction in labor for a mother or sister

**Table 2.** Estimated medical and food expenses at time of interview on June 10, 2006 (US$1 = 1,845 shillings).

**Item**	**Amount (Ugandan shillings)**	**Amount in US $**

HIV testing (Blood test)	4,000	2.17

Transportation to clinic (roundtrip) – required 2 to 3 times weekly	1,400	0.76

Traditional medicine (over duration of illness)	50,000 to 75,000	27.10 to 40.65

Non-traditional medicine	Unknown	Unknown

Foods recommended for consumption by persons with HIV		
Meat	2,800 / kg	1.52 / kg
Peas	2,000 / kg	1.08 / kg
Sugar	1,800 / kg	0.98 / kg
Rice	1,000 / kg	0.54 / kg
Passion fruit juice	1,000 / liter	0.54 / liter
Pawpaw (papaya)	800 / each	0.43 each
Irish potatoes	500 / 5 kg bag	0.27 / 5 kg
Milk	400 / liter	0.22 /liter
Cabbage	300 / each	0.16 each
Sweet bananas	300 / bunch	0.16 / bunch
Mango	50 to 200 / each	0.03 to 0.11 each
Fish	100 / kg	0.05 / kg
Eggs	100 / dozen	0.05 / dozen

Transportation of body after death	30,000 to 40,000	16.26 to 21.68

Funeral	50,000	27.10

**Table 3.** Average monthly food quantities (kg) received by 23 members of TASO, a community-based AIDS support group.

**Food Type**	**Minimum amount (kg)**	**Maximum amount (kg)**	**Mean amount (kg)**

Posho (corn meal)	10	25	23.3
Soy	12	25	21.7
Cowpeas	5	20	11.6
Beans	3	17.5	9.5
Cooking oil	3	4	3.5

## Appendix A: Interview Guide

- Household Composition
  ○ What changes in the structure of the households in your community have you seen in the last few years?
  ○ Have family members left households (for example, because of school attendance, migration, marriage, or death)? What role did the members that left play in the household?
  ○ Have households taken in additional family members (for example, returning schoolchildren, orphans, or widows)? What role do these new members play in the new household?
- Labor Allocations
  ○ When members of a household move to another place, do they generally send money back to the household?
  ○ What is the approximate length of a working day (in hours)? How much of the working time is allocated for agricultural or non-agricultural activities? Have working hours changed over the past 5 years?
  ○ How do labor contributions vary among the members of a household?
  ○ If a person in your community is not feeling well, does he or she continue to work? If yes, how many hours will a sick person work?
  ○ Who provides care for a sick family member?
  ○ What is the relationship of the caretakers to the person sick in the family?
- Land Transactions
  ○ How and when is land transferred between households?
- Health Expenditures
  ○ What are the different sources through which people receive health care in your community? What are the relative costs?
  ○ When a household member is sick, what increased expenditures does the household incur during illness?
  ○ What expenses incurred after a loss of a family member?
  ○ Are there households in your community who have difficulty meeting their expenses? If so, what ways do they find to meet expenses?
  ○ Do families get any support from other relatives in form of money, or activities in their farms?
- Cropping Patterns
  ○ How does this year’s harvest compare with last year’s harvest?
  ○ What has been the production level for the last 5 years?
  ○ Is the focus on crops grown on cash crops or food crops? Have you seen a shift to crops that require less labor or fewer other inputs?
  ○ Have you noticed any changes in cropping patterns and choices in the past 5 years?
- Agricultural Practices
  ○ How much land area was cultivated last year, and how much has been cultivated this year?
  ○ What changes have you observed over the past 5 years in terms of crop choices, areas under cultivation (in acres or hectares), and adoption of new technologies?
  ○ What is the role of the Agricultural Extension Services in your community?
- Market Participation
  ○ Do members of households in your community work for other households? If so, is this work in-kind or for wages?
  ○ Have you seen changes in agricultural wage rates in your community in recent years?
  ○ Is there anything else you would like to tell me about the economic and/or farming systems in this community?
